# Association of Serum Glucose/Potassium Ratio with Injury Severity and Transfusion Requirements in Traumatic Pelvic Fractures: A Retrospective Cohort Study

**DOI:** 10.3390/diagnostics16060939

**Published:** 2026-03-22

**Authors:** Abdullah Alper Sahin, Yunus Emre Özbilen, Çağrı Akalın

**Affiliations:** 1 Department of Orthopedics and Traumatology, Faculty of Medicine, Ordu University Training and Research Hospital, 52200 Ordu, Türkiye; dr.a.alpersahin@gmail.com (A.A.S.); yunusemreozbilen@hotmail.com (Y.E.Ö.); 2 Department of General Surgery, Faculty of Medicine, Ordu University, 52200 Ordu, Türkiye

**Keywords:** pelvic fracture, glucose-to-potassium ratio, transfusion, injury severity score, trauma, metabolic stress marker

## Abstract

**Background:** We evaluated the association between admission serum glucose-to-potassium ratio (GPR) and injury severity as well as early transfusion requirements in patients with traumatic pelvic fractures. **Methods:** This single-center, retrospective cohort study included 84 adult patients with isolated or predominantly pelvic fractures admitted between January 2020 and December 2024. Patients with concomitant non-pelvic skeletal fractures were excluded to isolate the metabolic response attributable to pelvic injury. GPR was calculated from admission serum glucose and potassium levels. Higher transfusion requirement (HT) was defined as ≥4 units of packed red blood cells within 24 h. Receiver operating characteristic (ROC) analysis identified the optimal GPR cut-off using the Youden index. Internal validation was performed using bootstrap resampling (1000 iterations), and model calibration was assessed with the Hosmer–Lemeshow test. The incremental discriminatory value of GPR beyond the Injury Severity Score (ISS) was evaluated by comparing AUC values using the DeLong test, and reclassification metrics including the category-free net reclassification improvement (NRI) and integrated discrimination improvement (IDI) were calculated. Sensitivity analyses were conducted using alternative transfusion thresholds (≥6 and ≥10 units). **Results:** The optimal GPR cut-off was 34 (area under the curve (AUC) = 0.730; 95% CI: 0.593–0.853; sensitivity 78.8%; specificity 59.0%). Patients with GPR ≥ 34 (*n* = 43) had significantly higher ISS values (median 25 [IQR: 16–34] vs. 9 [5–17]; *p* < 0.001), greater transfusion volumes (median 3 [0–6] vs. 0 [0–1] units; *p* < 0.001), and longer intensive care unit (ICU) stays (3 (0–6) vs. 0 (0–1) days; *p* < 0.001). In univariable logistic regression, GPR was significantly associated with HT (OR = 1.059 per unit increase; 95% CI: 1.015–1.104; *p* = 0.008); however, significance was not retained in the multivariable model after adjustment for ISS (*p* = 0.194). ISS remained the sole independent predictor (OR = 1.128; *p* < 0.001). The combined ISS + GPR model yielded an AUC of 0.857, representing a modest increment over ISS alone (AUC = 0.849; ΔAUC = 0.009; DeLong *p* = 0.566). Bootstrap-corrected AUCs confirmed minimal optimism (GPR alone: 0.726; ISS + GPR: 0.847). The Hosmer–Lemeshow test indicated adequate calibration for all models (*p* > 0.05). The category-free NRI was 0.627 (*p* = 0.009), whereas the IDI did not reach significance (0.017; *p* = 0.290). Sensitivity analysis at the ≥6-unit threshold yielded consistent results (GPR AUC = 0.709). **Conclusions:** Admission GPR is significantly associated with injury severity, hemorrhagic burden, and transfusion requirements in patients with traumatic pelvic fractures. Although GPR does not independently predict transfusion needs beyond ISS, it yields significant reclassification improvement and may serve as a practical, rapidly obtainable adjunct for early risk stratification in the acute trauma setting. Level of Evidence: III (retrospective prognostic study).

## 1. Introduction

Pelvic fractures are serious injuries that most commonly result from high-energy trauma and can be life-threatening in the early period [1]. These patients have significantly elevated mortality rates due to both the development of hemorrhagic shock secondary to massive blood loss and the concomitant organ injuries that frequently accompany pelvic ring disruptions [2]. The window for intervention is narrow. The severity of pelvic fractures varies over a wide range; while stable fractures generally have a favorable clinical course, unstable fractures carry a high risk of massive hemorrhage, hemodynamic instability, and death [3]. Early identification of patients at high risk for clinical deterioration is therefore of critical importance for timely initiation of damage-control resuscitation and definitive management.

Serum glucose and potassium levels are fundamental biochemical markers reflecting systemic metabolic status and are widely used parameters in clinical practice. Hyperglycemia, which develops as a physiological response to trauma and acute stress, has been associated with increased mortality and morbidity [4,5]. Hypokalemia, by contrast, may occur because of catecholamine-mediated intracellular potassium shift and sympathoadrenal activation [6,7]. The serum glucose-to-potassium ratio (GPR) is a composite index reflecting the integrated metabolic stress response, and previous studies have established its prognostic significance in aneurysmal subarachnoid hemorrhage (SAH), intracerebral hemorrhage, traumatic brain injury, and carbon monoxide poisoning [8,9,10,11].

This study was designed based on the hypothesis that elevated admission GPR values would be associated with greater anatomical Injury Severity Score (ISS) and increased hemorrhagic burden (higher transfusion requirements). The primary aim was to evaluate whether admission serum GPR is associated with injury severity and early transfusion requirements in patients with traumatic pelvic fractures.

## 2. Materials and Methods

### 2.1. Study Design and Setting

This was a single-center, retrospective cohort study. Adult patients who presented to our institution due to trauma and were diagnosed with pelvic fractures between January 2020 and December 2024 were evaluated. The study was reported in accordance with the Strengthening the Reporting of Observational Studies in Epidemiology (STROBE) guidelines [12].

### 2.2. Participants

Patients aged ≥ 18 years with at least 30 days of clinical follow-up data were included. The exclusion criteria were as follows: (1) age < 18 years; (2) anticoagulant use, steroid-related medication use, or diabetes mellitus; (3) renal dysfunction, defined as serum creatinine > 1.5 mg/dL or estimated glomerular filtration rate < 60 mL/min/1.73 m^2^; (4) prior potassium supplementation or insulin administration before blood sampling; (5) concomitant major vascular injury requiring emergency surgical repair; (6) concomitant non-pelvic skeletal fractures (to isolate the metabolic stress response attributable to pelvic injury and to minimize confounding from additional musculoskeletal injury on both ISS composition and GPR values); (7) in-hospital mortality within 24 h (precluding adequate transfusion assessment); and (8) incomplete laboratory or clinical data.

The local ethics committee of the institution approved the study protocol (No: 2025/285; approved on 12 September 2025), and written informed consent was obtained from all patients or their legal representatives. The study was performed in accordance with the ethical standards laid down in the Declaration of Helsinki.

### 2.3. Data Collection

Demographic characteristics (age, sex), mechanism of injury, energy level of the injury, ISS values, Tile classification of the pelvic fracture, treatment modality (surgical/conservative), transfusion volumes, intensive care unit (ICU) and hospital lengths of stay, and 30-day mortality outcomes were obtained from hospital electronic medical records. Serum glucose and potassium levels measured in venous blood samples obtained at the time of admission were recorded, and the GPR was calculated as the ratio of glucose (mg/dL) to potassium (mEq/L). Higher transfusion requirement (HT) was defined as transfusion of ≥4 units of packed red blood cells within the first 24 h of admission. This threshold captures clinically significant hemorrhage below the conventional massive transfusion (MT) criterion (≥10 units/24 h). It corresponds to the critical administration threshold component (>4 units within 1 h) described by Wei et al. in multiple trauma [13] and falls within the range associated with adverse outcomes in hemodynamically unstable pelvic fractures, where survivors received a mean of 6.0 units versus 11.6 among non-survivors [14]. The robustness of this definition was tested through sensitivity analyses at ≥6 and ≥10 units (Section 3.6).

### 2.4. Statistical Analysis

Statistical analyses were performed using IBM SPSS Statistics (version 27.0; IBM Corp., Armonk, NY, USA) and MedCalc Statistical Software (version 22.0; MedCalc Software Ltd., Ostend, Belgium). We assessed normality using the Shapiro–Wilk test. Normally distributed data were expressed as mean ± standard deviation, whereas non-normally distributed data were reported as median (interquartile range [IQR]). Categorical variables are reported as counts and percentages. We compared non-normally distributed continuous variables between groups using the Mann–Whitney U test, and categorical variables were compared using Fisher’s exact test or the chi-square test, as appropriate. We calculated Spearman’s rank correlation coefficients to evaluate bivariate associations between GPR and clinical outcome variables. Receiver operating characteristic (ROC) curve analysis was performed to assess the discriminatory capacity of GPR for HT, and the optimal cut-off value was determined using the Youden index [15]. Univariable and multivariable logistic regression analyses were conducted to evaluate associations with HT. We constructed multivariable models in a hierarchical fashion: a primary model including GPR and ISS; an extended model additionally incorporating age, sex, pelvic instability, and admission hemoglobin (HGB_0_) to assess the independent association of GPR after adjustment for available clinical confounders. Serum lactate was available in only 34 of 84 patients (40.5%) and was therefore not included in the primary multivariable models; a subgroup sensitivity analysis was performed in the lactate-available cohort. To quantify the incremental value of adding GPR to ISS, the following analyses were performed: (1) area under the curve (AUC) comparison between ISS-alone and ISS + GPR models using the DeLong test; (2) category-free net reclassification improvement (NRI); and (3) integrated discrimination improvement (IDI), following the methodology described by Pencina et al. [16]. Model calibration was assessed using the Hosmer–Lemeshow goodness-of-fit test. Bootstrap resampling (1000 iterations) was performed using the Harrell optimism-correction method to generate optimism-corrected AUC estimates.

Sensitivity analyses were performed by repeating the primary ROC and logistic regression analyses at alternative transfusion thresholds (≥6 and ≥10 units) to assess the robustness of the observed GPR–transfusion association. A complete case analysis was performed; patients with incomplete data were excluded prior to analysis. Given the exploratory nature of this study, no a priori sample size calculation was performed; however, the events-per-variable ratio (11.5; 23 events/2 variables) exceeded the minimum recommended threshold of 10 for logistic regression models. A two-tailed *p* < 0.05 was considered statistically significant. A generative artificial intelligence tool (Claude; Anthropic, 2025) was used solely for English language editing and grammatical refinement of the manuscript text. All study design, data collection, statistical analysis, interpretation, and conclusions were performed entirely by the authors without AI assistance.

### 2.5. Assessment of Bias

Several potential sources of bias were identified and addressed. Selection bias was minimized by including all consecutive patients meeting the eligibility criteria during the study period. To reduce information bias related to GPR measurement, only admission blood samples obtained prior to any therapeutic intervention (including fluid resuscitation and blood transfusion) were included in the analysis. However, the exact timing of blood sampling relative to the injury could not be standardized due to the retrospective design; at our institution, emergency blood sampling is typically performed within 15–30 min of emergency department arrival. Confounding was addressed by including ISS in the multivariable logistic regression model. Admission HGB_0_, available for all 84 patients on retrospective retrieval, was additionally incorporated as a covariate to assess its confounding potential; results are presented in Section 3.5. Serum lactate was retrospectively retrievable in only 34 of 84 patients (40.5%); this high proportion of missing data precluded its inclusion in primary models, but a subgroup sensitivity analysis was performed (Section 3.5). Residual confounding by unmeasured variables (e.g., systolic blood pressure, heart rate, pre-hospital fluid administration) cannot be excluded, as these parameters were not systematically recorded.

## 3. Results

### 3.1. Baseline Characteristics

**Figure 1:**
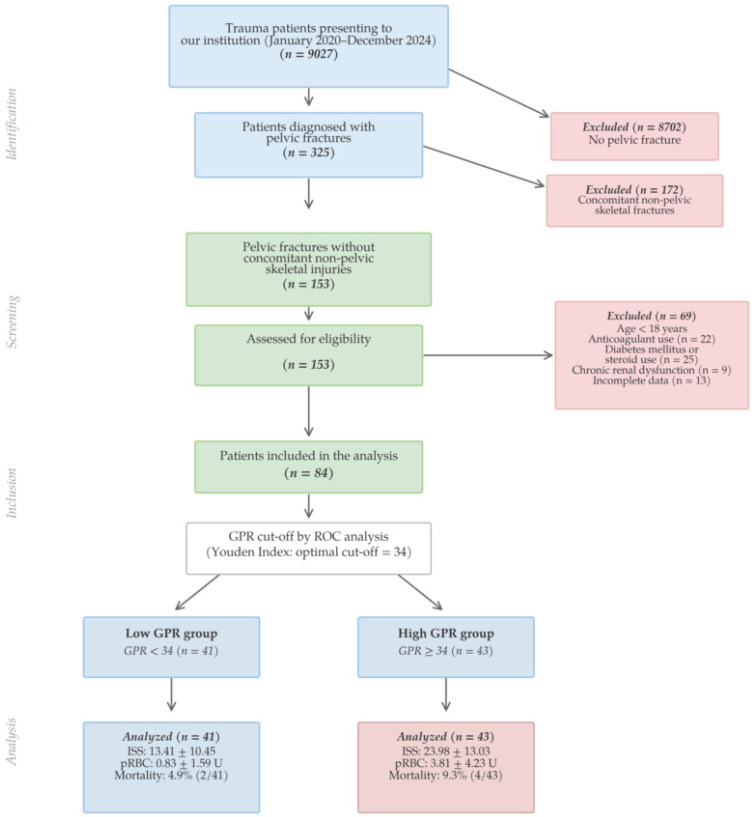
Flow diagram of patient selection. Patients with additional non-pelvic skeletal fractures were excluded; however, those with soft tissue injuries to other body regions may still be present in the final cohort.

The demographic data and clinical characteristics of the 84 patients included in the study are shown in Table 1. The patient selection process is summarized in Figure 1. The mean age was 50.04 ± 20.52 years, with 50 patients (59.5%) being male. The most common mechanisms of injury were falls from height (41.7%) and traffic accidents (39.3%). According to the Tile classification—selected for its direct role in guiding surgical decision-making at our institution—the majority of fractures were Type B (59.5%), followed by Type A (38.1%) and Type C (2.4%). Low-energy mechanisms (defined as falls from standing height or <1 m) accounted for 83.3% of injuries, in keeping with the demographic profile of a non-Level-I community hospital. Unstable pelvic fractures were identified in 52 patients (61.9%). Treatment was conservative in 48 patients (57.1%) and surgical in 36 (42.9%). The higher transfusion group comprised 23 patients (27.4%), while the remaining 61 patients (72.6%) received fewer than four units within 24 h. The overall 30-day mortality rate was 7.1% (*n* = 6).

### 3.2. ROC Analysis and GPR Cut-Off Determination

**Figure 2:**
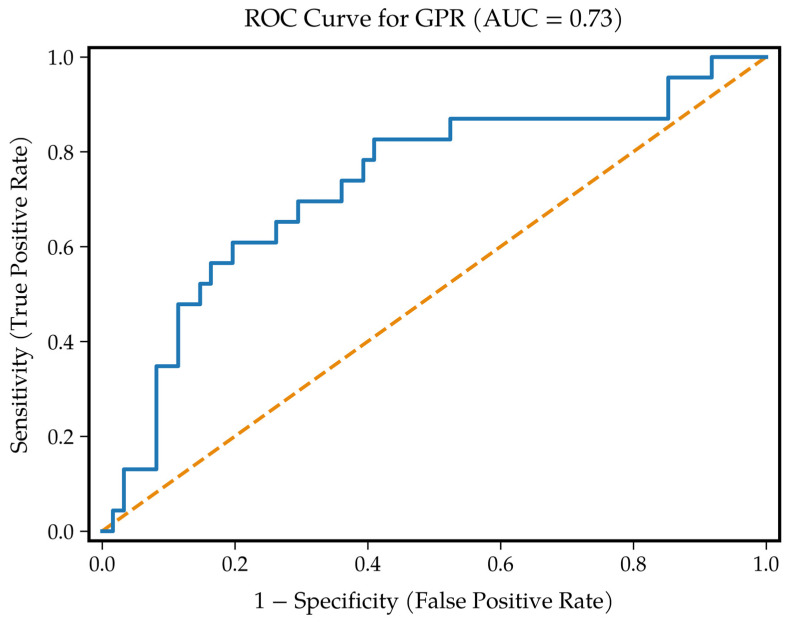
Receiver operating characteristic (ROC) curve of glucose-to-potassium ratio (GPR) for discriminating higher transfusion requirement. Area under the curve (AUC) = 0.730; 95% CI: 0.593–0.853.

ROC analysis yielded an AUC of 0.730 (95% CI: 0.593–0.853) for GPR against the higher transfusion outcome, reflecting acceptable discrimination for a single biochemical ratio (Figure 2). The Youden index placed the optimal cut-off at 34. At this threshold, sensitivity was 78.8% and specificity was 59.0%; the value was applied as a predefined binary classifier for all between-group comparisons.

### 3.3. Correlation Analysis

Spearman’s rank correlation analysis revealed moderate positive correlations between admission GPR and ISS (*ρ* = 0.465; *p* < 0.001), transfusion volume (*ρ* = 0.470; *p* < 0.001), and ICU length of stay (*ρ* = 0.421; *p* < 0.001). A weaker but statistically significant positive correlation was observed between GPR and total hospitalization duration (*ρ* = 0.371; *p* < 0.001).

### 3.4. Between-Group Comparisons

**Figure 3:**
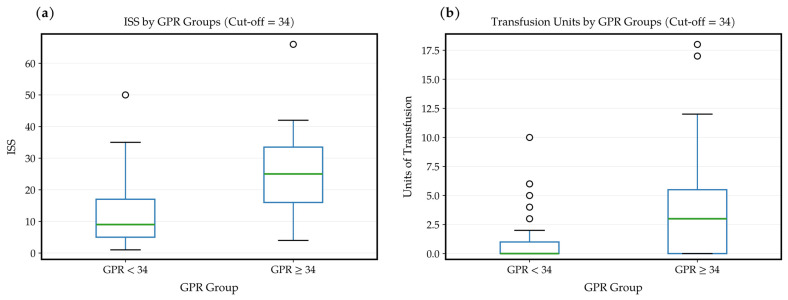
(**a**) Comparison of ISS Between Low and High GPR Groups, (**b**) Comparison of Blood Transfusion Requirements Between Low and High GPR Groups (Cut-off = 34).

When injury severity was compared between the GPR groups, patients with GPR ≥ 34 had significantly higher ISS values than those with GPR < 34 (25 (16–34) vs. 9 (5–17); *p* < 0.001). The requirement for blood transfusion was significantly greater in the high GPR group (3 (0–6) vs. 0 (0–1) units; *p* < 0.001). Higher transfusion rates (≥4 units/24 h) were significantly more common among patients with GPR ≥34 (41.9% vs. 12.2%; *p* = 0.003). The high GPR group had significantly longer hospitalization durations (9 (6–18) vs. 5 (3–10) days; *p* = 0.007) and ICU stays (3 (0–6) vs. 0 (0–1) days; *p* < 0.001). There was no significant difference in 30-day mortality between the two groups (*p* = 0.676) (Table 2, Figure 3).

### 3.5. Multivariable Logistic Regression

In univariable logistic regression analysis, GPR was significantly associated with HT (OR = 1.059 per unit increase; 95% CI: 1.015–1.104; *p* = 0.008). However, in the multivariable model including both GPR and ISS, only ISS retained independent significance (OR = 1.128; 95% CI: 1.062–1.198; *p* < 0.001), while the effect of GPR was attenuated (OR = 1.030; 95% CI: 0.985–1.076; *p* = 0.194). The multivariable model yielded good overall discrimination (AUC = 0.857) (Table 3).

To quantify the incremental contribution of GPR, three complementary approaches were applied: discrimination comparison, calibration assessment, and reclassification analysis. The ISS-alone logistic model yielded an AUC of 0.849; adding GPR increased this to 0.857, a non-significant increment (ΔAUC = 0.009; DeLong *p* = 0.566). Bootstrap internal validation confirmed minimal optimism: the corrected AUC was 0.850 for ISS alone and 0.847 for the combined model, indicating stable performance estimates. Hosmer–Lemeshow testing showed adequate calibration across all models—GPR alone (*χ*^2^ = 7.03, df = 8, *p* = 0.534), ISS alone (*χ*^2^ = 9.70, df = 8, *p* = 0.287), and the combined model (*χ*^2^ = 3.47, df = 8, *p* = 0.902)—with the combined model achieving the closest agreement between predicted and observed event rates. Reclassification analysis yielded a different picture. The category-free NRI was 0.627 (95% CI: 0.141–1.088; *p* = 0.009), reflecting improved classification of both events (NRI-events = 0.217) and non-events (NRI-non-events = 0.410). The IDI was 0.017 (95% CI: −0.014 to 0.048; *p* = 0.290) (Table 4).

To assess the confounding potential of baseline hematological status, admission hemoglobin (HGB_0_) was retrospectively retrieved and confirmed complete for all 84 patients (mean 13.18 ± 1.79 g/dL; median 13.15 [IQR 12.10–14.85] g/dL). HGB_0_ showed no significant correlation with GPR_0_ (Spearman *r* = −0.029; *p* = 0.795) or ISS (*r* = +0.091; *p* = 0.411). After co-adjustment for HGB_0_, GPR retained independent significance for HT (OR = 1.058; 95% CI: 1.015–1.103; *p* = 0.008), while HGB_0_ itself was not an independent predictor (OR = 0.933; 95% CI: 0.701–1.241; *p* = 0.63). The discriminatory performance of the GPR + HGB_0_ model was identical to GPR alone (AUC = 0.730), indicating that baseline anemia status does not confound the observed GPR–transfusion association. In the full three-variable model (ISS + GPR + HGB_0_), ISS remained the sole significant predictor (OR = 1.136; *p* < 0.001); both GPR (OR = 1.032; *p* = 0.173) and HGB_0_ (OR = 0.810; *p* = 0.25) were non-significant, consistent with the primary two-variable analysis.

To further evaluate whether GPR provides independent information beyond a broader set of clinical confounders, an extended multivariable model was constructed incorporating GPR, age, sex, pelvic instability, and HGB_0_ (without ISS). In this confounder-adjusted model, GPR retained independent significance (OR = 1.053; 95% CI: 1.009–1.098; *p* = 0.017), while age (*p* = 0.158), sex (*p* = 0.162), pelvic instability (*p* = 0.504), and HGB_0_ (OR = 0.755; 95% CI: 0.517–1.103; *p* = 0.146) were not independently associated with HT (model AUC = 0.741; Hosmer–Lemeshow *p* = 0.394). When ISS was additionally incorporated into this extended model, ISS remained the dominant predictor (OR = 1.142; 95% CI: 1.064–1.226; *p* < 0.001), and GPR was no longer significant (OR = 1.032; 95% CI: 0.983–1.082; *p* = 0.206), consistent with the primary two-variable analysis. The likelihood ratio test comparing the extended model with and without GPR confirmed that GPR did not significantly improve model fit after ISS adjustment (*χ*^2^ = 1.557; df = 1; *p* = 0.212). Of note, the incremental DeLong test and reclassification metrics remained stable after HGB_0_ adjustment: ISS + HGB_0_ versus ISS + GPR + HGB_0_ yielded ΔAUC = 0.014 (DeLong *p* = 0.323) and a category-free NRI of 0.627 (*p* = 0.010). Serum lactate, available in only 34 of 84 patients (40.5%), was evaluated in a subgroup sensitivity analysis. Patients with available lactate data had a higher mortality rate than those without (14.7% vs. 2.0%; Fisher *p* = 0.038), suggesting that lactate was preferentially measured in more severely injured patients. In the lactate-available subgroup, GPR retained significance in a biomarker-only model (GPR + HGB_0_ + lactate: GPR OR = 1.105; *p* = 0.040); however, the small sample size and high proportion of missing data preclude definitive conclusions.

### 3.6. Sensitivity Analyses

The association between GPR and transfusion requirement was further evaluated using alternative thresholds. At the ≥6-unit threshold (14 events, 16.7%), GPR yielded an AUC of 0.709, with an optimal cut-off of 39.3 (sensitivity 78.6%, specificity 67.1%) and a univariable OR of 1.048 per unit increase (95% CI: 1.008–1.136). The combined ISS + GPR model achieved an AUC of 0.864 at this threshold. At the ≥10-unit threshold, only 4 events (4.8%) were available, precluding reliable multivariable analysis; the GPR AUC was 0.684. The directionally consistent associations across thresholds support the consistency of the observed GPR–transfusion relationship (Table 5).

## 4. Discussion

The serum GPR, measured at hospital admission, was associated with injury severity and early hemorrhagic burden in patients with pelvic fractures. To the best of our knowledge, this is the first study to specifically evaluate the GPR in patients with pelvic trauma. Using a GPR cut-off value of 34 derived from transfusion requirement, patients could be stratified into two groups exhibiting significant differences in ISS, blood transfusion volumes, ICU and hospital lengths of stay. ISS, however, was clearly the dominant determinant. While GPR showed significance in the univariable analysis, the multivariable model revealed that ISS remained the sole independent predictor of HT. This finding is consistent with the hypothesis that GPR captures a complementary, overlapping dimension of injury severity rather than serving as a standalone prognostic marker. The data leave little doubt on this point. That said, the clinical value of a biomarker need not hinge on multivariable independence alone.

Although no previous work has specifically examined pelvic fractures in isolation, MT prediction in multiple trauma has received attention [13]. Wei et al., in their decision-tree analysis of MT risk in multiple trauma, identified systolic blood pressure, ISS, INR, and injury type as the principal determinants; shock index, heart rate, and hemoglobin also differed between transfusion groups in univariable analysis, though they did not appear in the final model. Metabolic biomarkers such as GPR were not evaluated [13].

### 4.1. GPR in the Context of Traumatic Injury

Smith et al. identified early predictors of mortality in hemodynamically unstable pelvic fractures [14]. Our findings complement this work by showing that GPR, a simple biochemical ratio, captures aspects of these injury severity dynamics, even if it does not emerge as an independent predictor once ISS is included in the model. Gabbe et al. examined 348 severe pelvic ring fractures in a population-based design and identified age ≥ 65 years (OR 7.55), field hypotension (OR 5.54), and severe thoracic injury (OR 2.81) as the dominant mortality predictors [2]. ISS itself was superseded by body-region-specific AIS scores in the final model—a finding that reinforces a broader point: no single variable, whether anatomical, physiological, or biochemical, is sufficient for comprehensive risk stratification in pelvic trauma.

Rommens et al. compared mortality rates according to fracture classification and found mortality rates of 5% in Type B fractures and 15% in Type C fractures [17]. In our study, mortality was observed predominantly in patients with Type B injuries, with an overall mortality rate of 7.1%. The low proportion of Type C fractures in our cohort (2.4%) limits comparisons for this subgroup but parallels the known epidemiological distribution at single-center, non-Level-I trauma referral institutions.

### 4.2. GPR as a Composite Metabolic Marker

The prognostic value of GPR has been examined across several non-pelvic trauma and vascular populations [18]. Zhou et al. found that GPR was significantly associated with both injury severity and mid-term prognosis in a large cohort of patients with acute traumatic spinal cord injury [18]. Similarly, Boyuk reported that GPR could differentiate between massive and non-massive pulmonary embolism [19], while Jung et al. confirmed the prognostic significance of the plasma GPR for mortality following aneurysmal SAH [20]. Katipoğlu and Demirtaş demonstrated that GPR had 72.7% sensitivity and 84.1% specificity for predicting mortality in blunt abdominal trauma [21], and Buz and Ustaalioğlu reported an AUC of 0.831 for GPR in predicting mortality following penetrating thoracic injuries [22]. Of note, Unal and Doğan found that GPR was not significantly associated with mortality in isolated blunt head trauma (*p* = 0.261), suggesting that the predictive capacity of GPR may vary across different trauma populations and injury patterns [23].

The contrast between the Unal and Doğan’s negative finding and the positive result reported by Shibata et al. in severe traumatic brain injury (GPR ≥ 50; adjusted OR = 4.079; *p* = 0.030) warrants attention. Shibata employed a composite endpoint encompassing death and vegetative state (Glasgow Outcome Scale 1–2); Unal restricted the outcome to mortality alone [23,24]. GPR, it appears, may be more sensitive to overall neurological deterioration than to mortality per se when intracranial pathology dominates. Indeed, the reproducibility of the GPR signal across independent cohorts is striking. In aneurysmal SAH, both Fujiki et al. [8] and Jung et al. [20] confirmed GPR as a significant multivariable predictor (OR 1.070; *p* < 0.001 in the Jung cohort) despite differing enrollment windows and outcome definitions—a degree of cross-study reproducibility that is uncommon for a single-laboratory biomarker. Similarly, Boyuk demonstrated GPR independence for differentiating massive from non-massive pulmonary embolism (OR 1.114; *p* = 0.011) [19]—a non-traumatic setting in which stress operates through right ventricular strain rather than direct tissue injury. That the same ratio retains discriminatory value across such disparate contexts strengthens the biological case for GPR.

The GPR has been evaluated under the designation ‘Stress Index’ (SI) as an indicator of metabolic derangement across several trauma populations [24,25,26]. The evidence spans distinct injury types. Huang et al. reported that the SI was an independent risk factor for in-hospital mortality following traumatic femoral fractures (adjusted OR 2.05; *p* = 0.016), achieving an AUC of 0.609 [26]. Taniguchi et al. reported an AUC of 0.80 for severe trauma prediction (ISS ≥ 16) and—unusually for the GPR literature—validated the model prospectively (r = 0.70) [25]. In that cohort, SI was the sole laboratory variable significantly associated with all three clinical endpoints (severe trauma, damage-control surgery, and MT), outperforming heart rate, systolic blood pressure, lactate, and hematocrit individually [25]. This is a notable finding. Across these populations, ROC-derived GPR cut-offs cluster in the 26–40 range (mg/dL-based), with AUCs spanning 0.609 to 0.842. Wu et al. reported a comparable AUC of 0.720 in intracerebral hemorrhage, where GPR retained multivariable independence (adjusted OR 2.945; *p* = 0.032) [9]. Our cut-off of 34 and AUC of 0.730 sit squarely within this range. The convergence with Demirtaş et al. in carbon monoxide poisoning (35.9; AUC = 0.791) [11] and Katipoğlu and Demirtaş in blunt abdominal trauma mortality (33.95; AUC = 0.771) [21] is striking—these studies evaluated entirely different pathologies, yet the thresholds cluster within a narrow band.

The outcome-dependence of the cut-off deserves emphasis. Katipoğlu and Demirtaş, who reported two distinct thresholds within the same blunt abdominal trauma cohort—33.95 for mortality and 26.15 for surgical indication (AUC = 0.709)—demonstrated that the optimal discriminatory threshold shifts with the clinical question being asked [21]. Of note, GPR retained multivariable independence in several of these studies—including spinal cord injury (adjusted for sex, injury level, and white blood cell count) [18], femoral fracture (adjusted for comorbidities, GCS, and ISS) [26], intracerebral hemorrhage (adjusted for GCS and hematoma volume) [9], and carbon monoxide poisoning (OR = 1.135; *p* < 0.001) [11]—In our cohort, it did not survive adjustment for ISS alone. The most plausible explanation is threefold: differences in outcome definition (mortality or functional recovery versus transfusion requirement), model composition, and the degree to which ISS subsumes the variance captured by GPR when the study population is restricted to predominantly pelvic injuries.

### 4.3. Incremental Value and Clinical Interpretation of AUC

Does GPR add discriminatory information beyond anatomical injury scoring? The ISS-alone model yielded an AUC of 0.849 for HT; combining GPR with ISS produced an AUC of 0.857, a difference that did not reach statistical significance (ΔAUC = 0.009; DeLong *p* = 0.566). By this conventional measure, GPR does not improve upon ISS. The distinction between discrimination and reclassification is relevant here [16,27]. While the AUC captures overall ranking performance across the entire threshold spectrum, reclassification indices (i.e., NRI and IDI) detect shifts in individual predicted probabilities that may be clinically meaningful even when the global AUC changes little [27]. In our data, the category-free NRI was 0.627 (95% CI: 0.141–1.088; *p* = 0.009), indicating that GPR reclassified a substantial proportion of patients in clinically appropriate directions—both among those who received high transfusion (NRI-events = 0.217) and those who did not (NRI-non-events = 0.410). The IDI, which quantifies the mean change in predicted probability between events and non-events, was modest and non-significant (0.017; *p* = 0.290). This pattern—significant reclassification with a non-significant discrimination increment—is not uncommon in biomarker research [27] and reflects a well-documented property of NRI: its sensitivity to directional probability shifts at the individual level exceeds that of the C-statistic. Pencina et al. showed that even negligible C-statistic increments can yield large and significant NRI values when individual risk estimates move in clinically appropriate directions [16]. The Hosmer–Lemeshow test confirmed adequate calibration for the combined model (*χ*^2^ = 3.470, df = 8, *p* = 0.902). This suggests that GPR improves the agreement between predicted and observed transfusion rates—a property distinct from discrimination that is often overlooked in biomarker evaluation [27]. From a practical standpoint, GPR carries an advantage that anatomical severity scoring does not: it is calculable within minutes of emergency department arrival from routine biochemistry, before imaging-based ISS determination becomes available.

In practice, this temporal advantage matters. Current pelvic trauma algorithms target hemorrhage control within 30 min of presentation; during this interval, assessment relies on physical examination, pelvic radiography, and focused assessment with sonography for trauma ultrasonography [3]. Base deficit exceeding 10 is the strongest laboratory predictor of mortality in this setting [3], yet arterial blood gas analysis is not universally available on arrival. GPR requires only routine venous biochemistry. It could, therefore, serve as an early metabolic triage signal during the narrow window before definitive imaging is completed. An AUC of 0.730 for GPR alone falls within the range considered acceptable for a single biochemical marker—comparable to the 0.609 reported by Huang et al. for the SI in femoral fracture mortality [26]. Recent data from Sharma et al. further indicate that potassium imbalances, particularly hypokalemia, significantly affect outcomes in patients with severe traumatic brain injury [28], supporting the biological basis for incorporating the potassium axis into composite stress indices.

### 4.4. Pathophysiological Considerations

The relationship between elevated GPR and hemorrhagic burden in trauma may be understood through the lens of the systemic stress response. Trauma-induced sympathoadrenal activation triggers a cascade of neuroendocrine events: catecholamine release—chiefly epinephrine and norepinephrine—promotes hepatic glycogenolysis and gluconeogenesis through direct stimulation of α- and β-adrenergic receptors on hepatocytes, while cortisol further amplifies hepatic glucose output and reduces peripheral insulin sensitivity [4,5]. The resulting stress hyperglycemia is proportional to the magnitude of tissue injury and blood loss. Concurrently, catecholamines stimulate β2-adrenergic receptor-mediated transcellular potassium uptake through activation of the Na^+^/K^+^-ATPase pump, driving potassium from the extracellular to the intracellular compartment [6,7]. Brown et al. quantified this pathway directly: physiological epinephrine infusion (0.05 μg/kg/min) lowered plasma potassium by 0.82 ± 0.19 mEq/L, an effect selectively abolished by β2-receptor antagonism but unaffected by β1-blockade [7]. This catecholamine-induced hypokalemia is further augmented by insulin secretion (stimulated by hyperglycemia itself) and by the alkalotic milieu that may arise during the early hyperventilatory response to hemorrhagic shock. The relationship is bidirectional. Rowe et al. showed that even mild-to-moderate potassium depletion impairs pancreatic beta-cell sensitivity, blunting insulin secretion and exacerbating glucose intolerance (r = 0.78 between total body potassium change and insulin response; *p* < 0.05) [6]. This mechanism is not trivial. The result is a self-amplifying loop: catecholamine-driven hypokalemia impairs insulin-mediated glucose disposal, which compounds the hyperglycemic numerator of the GPR. In the setting of severe hemorrhage, tissue hypoperfusion generates metabolic acidosis with lactate accumulation; the resulting acid–base disturbance triggers compensatory transcellular potassium redistribution that further modulates the potassium component of the GPR [8,9,10,11]. The clinical weight of this potassium axis is substantial. Sharma et al. reported extreme hypokalemia (<2.5 mEq/L) in 91.95% of patients at the time of death following severe traumatic brain injury [28]. Thus, GPR integrates at least three distinct physiological axes—sympathoadrenal activation, glucoregulatory disruption, and acid–base perturbation—each of which independently scales with injury severity.

### 4.5. Methodological Considerations

The practice of deriving and applying a diagnostic threshold within the same dataset warrants scrutiny. In our analysis, the GPR cut-off of 34 was identified through ROC optimization for HT and subsequently used to define comparison groups for this same outcome. This circularity does not invalidate the between-group differences, but it does mean that the apparent discriminatory performance may overestimate what would be observed in an independent cohort. Taken at face value, the non-significant ΔAUC could suggest that GPR adds nothing to ISS. We addressed this through bootstrap resampling (1000 iterations) to estimate the degree of optimism—defined as the difference between the AUC obtained when a model is tested on its training data versus new data [27]. The optimism was 0.004 for GPR alone (apparent 0.730 → corrected 0.726) and 0.010 for the combined model (apparent 0.857 → corrected 0.847). These values are small. They indicate that overfitting contributed minimally to the observed performance, a finding that accords with the adequate events-per-variable ratio of 11.5 in our logistic models.

A related consideration is the use of dichotomized GPR for group comparisons versus continuous GPR in regression analyses. Dichotomization at a data-driven threshold discards information and creates the impression of a binary risk when, in reality, the relationship between GPR and transfusion requirement is graded [27]. For this reason, continuous GPR served as the primary predictor in all regression and reclassification analyses presented here. The cut-off of 34 is offered as a clinical reference point—not as a validated decision threshold—and should be interpreted with the understanding that the empirical Youden method exhibits greater bias and variance in small samples than kernel-based or parametric alternatives [15]. The minimal optimism on bootstrap correction (0.004 for GPR alone) suggests, however, that this limitation did not materially inflate the reported performance. Prospective validation in an independent cohort would be required before any clinical implementation could be considered. The choice of ≥4 units as the higher transfusion threshold, while lower than the conventional MT criterion of ≥10 units, was selected to capture clinically meaningful hemorrhage in a single-system injury population where massive transfusion events are uncommon; the consistency of GPR associations across ≥4, ≥6, and ≥10 unit thresholds in our sensitivity analyses (Section 3.6) supports the robustness of this definition. This limitation is not unique to our study. The majority of published GPR analyses rely on single-center retrospective designs without external validation; among the existing literature, only Taniguchi et al. incorporated a prospective validation cohort [25].

Pelvic trauma management continues to evolve along multiple axes. Imaging-based innovations, including computed tomography–based computational modeling for sacroiliac screw placement [29], illustrate a broader shift toward multimodal injury assessment. Parallel refinements in ISS-based severity grading [30] confirm that anatomical scoring itself is not static. Within this evolving context, biochemical indices such as GPR may complement—rather than compete with—established tools by providing early metabolic information before definitive imaging becomes available.

### 4.6. Limitations and Future Directions

We recognize several limitations that constrain the interpretation of these findings. The retrospective, single-center design limits generalizability, and the sample size of 84 patients with 23 events afforded limited statistical power for detecting modest independent effects of GPR after ISS adjustment. The exclusion of 172 patients with concomitant non-pelvic skeletal fractures—53% of the initial pelvic fracture cohort—is a sobering reminder that our findings apply to a selected subset. Had individual-level data for these excluded polytrauma patients been available, a sensitivity analysis would have strengthened our conclusions considerably. This restriction narrows the study population in ways that may not reflect the broader polytrauma setting in which GPR might ultimately be applied. The practice of restricting the study population to isolated or predominantly single-system injuries is, however, not unique to our cohort. Zhou et al. applied an analogous exclusion in their spinal cord injury analysis, removing patients with AIS ≥ 3 in non-spinal body regions to isolate the metabolic response attributable to the index injury [18].

A further limitation concerns the completeness of physiological covariates. Admission hemoglobin (HGB_0_) was retrospectively retrieved and found to be complete for all 84 patients (mean 13.18 ± 1.79 g/dL; median 13.15 [IQR 12.10–14.85] g/dL). To assess its potential confounding, GPR was re-entered into a logistic model co-adjusted for HGB_0_: GPR retained significance (OR = 1.058; 95% CI: 1.015–1.103; *p* = 0.008), whereas HGB_0_ itself was not an independent predictor (OR = 0.933; *p* = 0.63). In the full ISS + GPR + HGB_0_ model, ISS remained the sole dominant independent predictor (OR = 1.136; *p* < 0.001); GPR (*p* = 0.173) and HGB_0_ (*p* = 0.25) were both non-significant, consistent with the primary two-variable model. An extended confounder-adjusted model incorporating age, sex, pelvic instability, and HGB_0_ confirmed that GPR retained significance in the absence of ISS (OR = 1.053; *p* = 0.017) but not after ISS adjustment (*p* = 0.206); these findings are detailed in Section 3.5. Serum lactate was available in only 34 of 84 patients (40.5%) and could not be reliably incorporated into primary multivariable analysis. A subgroup sensitivity analysis in the lactate-available cohort showed that GPR remained significant in a biomarker-only model (OR = 1.105; *p* = 0.040); however, the high rate of missing data and the selection bias inherent in lactate ordering (mortality 14.7% in the lactate-available group vs. 2.0% in the remainder; *p* = 0.038) preclude definitive conclusions. Systolic blood pressure, heart rate, GCS, and base deficit were not systematically archived in this retrospective dataset and remain unmeasured confounders. Their absence means that the univariable GPR–transfusion association may partly reflect general hemodynamic derangement rather than an independent metabolic signal—a distinction that only a prospective study incorporating hemodynamic and metabolic predictors alongside GPR could resolve.

Patients who died within 24 h of admission were excluded by protocol, which may introduce survivorship bias by removing the most severely injured patients from the analytic denominator. The six patients who died during the study period had a mean ISS of 42.7 and a mean GPR of 44.6, with five of six meeting the high-transfusion criterion (mean pRBC: 7.5 units). Their clinical profiles suggest that including early deaths would have strengthened, rather than attenuated, the observed GPR–transfusion association. The exact timing of venous blood sampling relative to injury could not be standardized across patients; because GPR reflects a dynamic physiologic stress response, time-dependent variability in sampling may have introduced measurement noise that would tend to bias results toward the null. Pre-injury nutritional intake and concurrent medications may also influence serum glucose and potassium concentrations, yet these variables could not be ascertained in the current dataset. Although patients with concomitant non-pelvic skeletal fractures were excluded, those with soft tissue injuries to other body regions remained in the cohort; consequently, elevated ISS values in some patients may partly reflect injuries beyond the pelvic ring. The absence of arterial blood gas data (base excess) and the shock index precludes a direct comparison between GPR and established markers of hemorrhagic shock.

The use of complete case analysis, whereby patients with missing data were excluded rather than handled through multiple imputation or inverse probability weighting, may have introduced bias if the missing data mechanism was not completely at random; the proportion and pattern of missingness were not formally tested. The exclusion of patients with diabetes mellitus, anticoagulant use, and renal dysfunction—populations frequently encountered in emergency departments—restricts the external validity of these findings and limits direct applicability to unselected trauma cohorts. Of the 84 patients in our cohort, 83.3% sustained low-energy injuries and only two (2.4%) had Tile Type C fractures—a case mix typical of a non-Level-I community hospital rather than a major trauma referral center. This case mix represents an important limitation. The clinical scenarios in which rapid metabolic triage would matter most—high-energy pelvic ring disruptions with hemodynamic instability—are underrepresented in our data. Whether the GPR cut-off of 34 retains its accuracy in that higher-acuity population remains untested and would require validation at Level-I trauma centers where such injuries are treated routinely. Future studies should adopt prospective, multicenter designs with larger sample sizes to determine whether serial GPR measurements and dynamic changes in this ratio provide additional prognostic information beyond a single admission value.

## 5. Conclusions

Admission GPR is associated with injury severity and early transfusion requirements in patients with traumatic pelvic fractures. Patients with GPR ≥ 34 had higher ISS values, greater transfusion volumes, and longer ICU and hospital stays than those below this threshold. GPR did not retain independent significance after adjustment for ISS in the multivariable model. This is an important negative finding that should temper expectations. Granted, a non-significant multivariable result does not negate clinical utility altogether. In fact, the significant category-free NRI of 0.627 (*p* = 0.009) indicates that GPR shifts individual risk estimates in clinically appropriate directions for a meaningful proportion of patients—a property that the AUC comparison alone does not capture. We suggest that GPR may have a role as a complementary triage adjunct in the hyperacute phase of pelvic trauma resuscitation, when ISS has not yet been calculated. Its practical advantages are difficult to dismiss: GPR requires no additional laboratory processing, incurs no cost, and is available from the first set of admission bloods. We note that these attributes do not substitute for anatomical severity scoring or physiologic assessment, but they provide an early metabolic signal during the interval before definitive imaging is completed. Because low-energy mechanisms predominated in this cohort, our findings may not generalize to the high-energy, hemodynamically unstable pelvic trauma populations that would benefit most from early metabolic triage. The absence of hemodynamic parameters in this retrospective dataset represents a residual limitation. Prospective studies incorporating systolic blood pressure, heart rate, and lactate alongside GPR are needed to determine whether this ratio provides independent prognostic value beyond standard clinical parameters. Although admission hemoglobin was retrospectively retrieved and its confounding potential formally tested—yielding no material change in the primary findings—the lack of hemodynamic variables limits causal inference. Multicenter designs with larger and more heterogeneous cohorts, serial GPR measurements, and external validation would be required before any clinical implementation could be considered.

## Figures and Tables

**Table 1:** Baseline Characteristics of the Study Population.

Variables	Total (*n* = 84)
Age, years (mean ± SD)	50.04 ± 20.52
Sex, *n* (%)	
Female	34 (40.5%)
Male	50 (59.5%)
Mechanism of injury, *n* (%)	
Traffic accident	33 (39.3%)
Fall from height (≥1 m or ≥5 steps)	35 (41.7%)
Crush/compression	2 (2.4%)
Other (agricultural accident, heavy object)	14 (16.7%)
Mechanic energy	
Low	70 (83.3%)
High	14 (16.7%)
ISS (%)	
1–15	35 (41.7%)
16–24	20 (23.8%)
≥25	29 (34.5%)
Unstable pelvis, *n* (%)	52 (61.9%)
Tile classification, *n* (%)	
Type A	32 (38.1%)
Type B	50 (59.5%)
Type C	2 (2.4%)
Treatment	
Conservative	48 (57.1%)
Surgery	36 (42.9%)
Blood transfusion (units, pRBCs)	
Low (<4 units/24 h)	61 (72.6%)
High (≥4 units/24 h)	23 (27.4%)
Hospitalization duration (days)	7 (4–14)
ICU stay (days)	1 (0–5)
Mortality, *n* (%)	6 (7.1%)

Data are presented as *n* (%) or mean ± standard deviation or median (IQR) as appropriate. ISS, Injury Severity Score; ICU, intensive care unit; SD, standard deviation; pRBCs, packed red blood cells.

**Table 2:** Comparison of Injury Severity and Transfusion Requirement Between Low and High GPR Groups.

	Low GPR (<34), *n* = 41	High GPR (≥34), *n* = 43	*p* Value
ISS	9 (5–17)	25 (16–34)	<0.001 *
Blood transfusion, units	0 (0–1)	3 (0–6)	<0.001 *
High transfusion (≥4 units), *n* (%)	5 (12.2%)	18 (41.9%)	0.003 **
Hospitalization duration (days)	5 (3–10)	9 (6–18)	0.007 *
ICU stay (days)	0 (0–1)	3 (0–6)	<0.001 *
Mortality	2 (4.9%)	4 (9.3%)	0.676 **

Data are presented as median (IQR) or number (%). ISS, Injury Severity Score; ICU, intensive care unit; GPR, glucose-to-potassium ratio. The GPR cut-off value (34) was derived based on higher transfusion requirement using ROC analysis and subsequently applied as a predefined threshold for group comparisons. * Mann–Whitney U test, ** Fisher’s exact test.

**Table 3:** Univariable and Multivariable Logistic Regression Analysis for Higher Transfusion Requirement.

Variable	Univariable			Multivariable		
	OR	95% CI	*p*	OR	95% CI	*p*
GPR (per unit)	1.059	1.015–1.104	0.008	1.030	0.985–1.076	0.194
ISS (per unit)	1.135	1.070–1.203	<0.001	1.128	1.062–1.198	<0.001

OR, odds ratio; CI, confidence interval; GPR, glucose-to-potassium ratio; ISS, Injury Severity Score. Dependent variable: higher transfusion requirement (≥4 units/24 h). Events-per-variable ratio: 11.5 (23 events/2 variables). Model AUC: 0.857. Hosmer–Lemeshow: *χ*^2^ = 3.470, *p* = 0.902.

**Table 4:** Incremental Discriminatory Performance of GPR Beyond ISS.

Metric	Value	95% CI	*p*
AUC—ISS alone	0.849	—	—
AUC—ISS + GPR	0.857	—	—
ΔAUC	0.009	—	0.566
Optimism-corrected AUC (ISS + GPR)	0.847	—	—
Category-free NRI	0.627	0.141–1.088	0.009
IDI	0.017	−0.014 to 0.048	0.290

AUC, area under the curve; NRI, net reclassification improvement; IDI, integrated discrimination improvement; GPR, glucose-to-potassium ratio; ISS, Injury Severity Score.

**Table 5:** Sensitivity Analysis: GPR Performance Across Transfusion Thresholds.

Threshold	Events, *n* (%)	GPR AUC	Cut-Off	Sensitivity	Specificity	ISS + GPR AUC
≥4 units (primary)	23 (27.4%)	0.730	34.0	78.8%	59.0%	0.857
≥6 units	14 (16.7%)	0.709	39.3	78.6%	67.1%	0.864
≥10 units	4 (4.8%)	0.684	46.0	75.0%	75.0%	— †

† Insufficient events for reliable multivariable modeling. AUC, area under the curve; GPR, glucose-to-potassium ratio; ISS, Injury Severity Score.

## Data Availability

The data presented in this study are available on request from the corresponding author due to privacy and ethical restrictions.

## Abbreviations

The following abbreviations are used in this manuscript:

AIS	Abbreviated Injury Scale
AUC	Area under the curve
CI	Confidence interval
df	Degrees of freedom
GCS	Glasgow Coma Scale
GPR	Glucose-to-potassium ratio
HGB_0_	Admission hemoglobin
HT	Higher transfusion requirement
ICU	Intensive care unit
IDI	Integrated discrimination improvement
IQR	Interquartile range
ISS	Injury Severity Score
MT	Massive transfusion
NRI	Net reclassification improvement
OR	Odds ratio
pRBC	Packed red blood cell
ROC	Receiver operating characteristic
SAH	Subarachnoid hemorrhage
SI	Stress index
STROBE	Strengthening the Reporting of Observational Studies in Epidemiology
